# Successful treatment of transplant-associated thrombotic microangiopathy with iptacopan: a non-adult case study

**DOI:** 10.3389/fimmu.2025.1583506

**Published:** 2025-08-13

**Authors:** Shijie Bao, Kaikai Huang, Xiao Huang, Huamin Zhu, Zhiqiang Sun

**Affiliations:** Shenzhen Hospital, Southern Medical University, Shenzhen, China

**Keywords:** iptacopan, complement, allo-HSCT, TA-TMA, factor B inhibitor

## Abstract

Transplant-associated thrombotic microangiopathy (TA-TMA) is a severe complication of hematopoietic stem cell transplantation (HSCT), characterized by microangiopathic hemolytic anemia, thrombocytopenia, microthrombosis, and multi-organ dysfunction. Mortality rates range from 50% to 90%, with higher rates observed in high-risk patients. The pathogenesis of TA-TMA involves abnormal activation of the complement system—particularly of the alternative pathway—resulting in endothelial injury and microthrombosis. We present the case of a 17-year-old man with high-risk TA-TMA who achieved a favorable outcome following the oral administration of the factor B inhibitor iptacopan. The patient exhibited significant improvements in laboratory markers, including reductions in lactate dehydrogenase, urine protein/creatinine ratio, and C5b-9 levels, along with recovery of platelet counts and haptoglobin levels. This case highlights the potential efficacy of iptacopan in the management of TA-TMA, particularly in high-risk patients, and suggests that complement factor B inhibition may offer a promising therapeutic strategy for this challenging condition.

## Introduction

Transplant-associated thrombotic microangiopathy (TA-TMA) is a serious complication of hematopoietic stem cell transplantation (HSCT), characterized by microangiopathic hemolytic anemia, thrombocytopenia, microthrombosis, and multi-organ dysfunction. The mortality rate among TA-TMA patients is estimated at 50% to 90%, with higher rates observed in high-risk individuals (1–3).

The pathophysiology of TA-TMA is complex, involving a cycle of endothelial cell activation that promotes a procoagulant state, along with the activation of antigen-presenting cells, lymphocytes, the complement cascade, and microthrombus formation. This has led to the formulation of the **“**Three-Hit Hypothesis**”**, in which patients with either an underlying predisposition to complement activation or pre-existing endothelial injury (Hit 1) undergo toxic conditioning regimens that exacerbate endothelial damage (Hit 2), followed by additional insults—such as medications, alloreactivity, infections, and/or antibodies (Hit 3) (4). The first-line treatment for TA-TMA focuses on identifying and removing the triggering cause and providing supportive care. This includes timely reduction or discontinuation of calcineurin inhibitors (CNIs)/rapamycin-targeted protein (mTOR) inhibitors, control of hypertension, and management of infections and graft-versus-host disease (GVHD). If the response to the first-line treatment is not satisfactory, second-line treatments, such as plasma exchange, eculizumab, rituximab, and difibrotide, are used in combination. Several studies have reported that Ba levels significantly increase in the early stages following allogeneic HSCT and are closely associated with the onset of TA-TMA (5–7). The alternative complement pathway (AP) plays a significant role in the pathogenesis of TA-TMA. In the activated AP, C3b is cleaved from C3 and attaches to an activated surface. Through a series of steps involving factor B, factor D, and factor P, the C3 and C5 convertases are formed. The C3 convertase amplifies the process by generating additional C3b, while the C5 convertase cleaves C5 into C5b, triggering the terminal complement pathway (7, 9).

Factor B serves as a central regulatory protein in this cascade. Factor D cleaves bound factor B into two fragments: Ba (a small fragment, released into the liquid phase) and Bb (a large fragment that remains bound to the complex) (7–9).

We report the case of a high-risk TA-TMA patient who achieved a favorable outcome following oral administration of the factor B inhibitor iptacopan—a therapeutic agent rarely used in TA-TMA to date.

## Case presentation

The patient is a 17-year-old man. In December 2023, he was diagnosed with pneumonia. Blood tests showed the following: a white blood cell count (WBC) of 3.25 × 10^9^/L, an absolute neutrophil count (NEUT#) of 1.10 × 10^9^/L, hemoglobin (HGB) of 77 g/L, and a platelet count (PLT) of 81 × 10^9^/L. Based on the morphology, immunology, cytogenetics, and molecular biology of bone marrow cells, the patient was diagnosed with myelodysplastic syndrome (MDS, +8, -7, IPSS-R 5.5 points, JAK2, ROBO2, and CROCC mutations). The patient is a teenager with high-risk MDS accompanied by poor prognostic genetic abnormalities and severe cytopenia, thus meeting the indications for bone marrow transplantation. On 16 July 2024, he received HLA-matched sibling donor allogeneic peripheral blood stem cell transplantation (MSD-PBSCT) (with pre-treatment BF regimen and GVHD prevention with CsA + MTX + MMF + ATG). Granulocyte reconstitution occurred on day +10 post-transplantation, and megakaryocyte reconstitution on day +14. Cyclosporine and mycophenolate mofetil were used for GVHD prevention.

Thirty-five days post-transplantation, the patient presented with dull pain in the lower central abdomen, along with diarrhea characterized by 4–6 episodes of yellow, watery stools per day. Urine output was reduced to 200–300 mL per day. No gross hematuria was observed. The patient exhibited a diminished mental state and complained of fatigue. Additional clinical parameters were as follows: WBC: 10.80 × 10^9^/L, NEUT#: 8.72 × 10^9^/L, HGB: 107 g/L, PLT: 27 × 10^9^/L; peripheral blood microscopy: schistocytes in approximately 1% (follow-up review 1%–2.5%). prothrombin time (PT): 20.3 s, prothrombin activity (PT%): 44%, activated partial thromboplastin time (APTT): 54.1 s, prothrombin time (TT): 23.0 s, fibrinogen (FIB): 1.3 g/L; alanine aminotransferase (ALT): 1379 U/L, aspartate aminotransferase (AST): 990 U/L, total bilirubin (TBil): 58.4 µmol/L, direct bilirubin (DBil): 37.5 µmol/L, indirect bilirubin (IBil) 20.9 µmol/L; lactate dehydrogenase (LDH) 615 U/L; creatinine (Cr) 592.5 µmol/L; direct anti-human globulin test (C3d) negative, direct anti-human globulin test (IgG) negative; haptoglobin (HAPT) <0.06 g/L; urinary microalbumin/creatinine ratio (ACR): 342.277 mg/g; ADAMTS13 result negative, C5b-9: 739 ng/mL (reference value: 75–219); CT scan of the chest and abdomen: (1) exudation in the lower lobes of both lungs and (2) uneven liver and spleen density, suggestive of hepatic impairment.

The patient had elevated LDH and urinary protein, new-onset thrombocytopenia and anemia, schistocytes in the peripheral blood, and elevated C5b-9. Given that TA-TMA is characterized by an elevated ACR (> 2mg/mg), significantly elevated C5b-9 (above the upper reference limit), and multi-organ dysfunction (acute renal failure, acute liver failure, and pulmonary infection), the patient was diagnosed with TA-TMA according to the above criteria.

On 20 August 2024, plasma exchange and daily hemodialysis were initiated and continued until 26 August. Eculizumab (900 mg) was administered once on 22 August. Given the patient’s critical condition, treatment was switched on Day 5 to the proximal complement inhibitor iptacopan (200 mg, twice daily) to achieve broader control over complement activation. As the patient had not been previously vaccinated against encapsulated bacteria, prophylactic antibiotics were also administered. The patient was found to have multiple infections. Epstein–Barr virus (EBV) DNA was detected on 26 August, and rituximab was administered once weekly for three doses. EBV DNA subsequently became undetectable in follow-up testing. Cytomegalovirus (CMV) DNA was detected on 29 August, and cidofovir was administered twice, after which CMV DNA also turned negative. In addition, sputum culture identified *Klebsiella pneumoniae*, and ceftriaxone was prescribed based on drug sensitivity results. The infection was brought under control.

**Figure 1:**
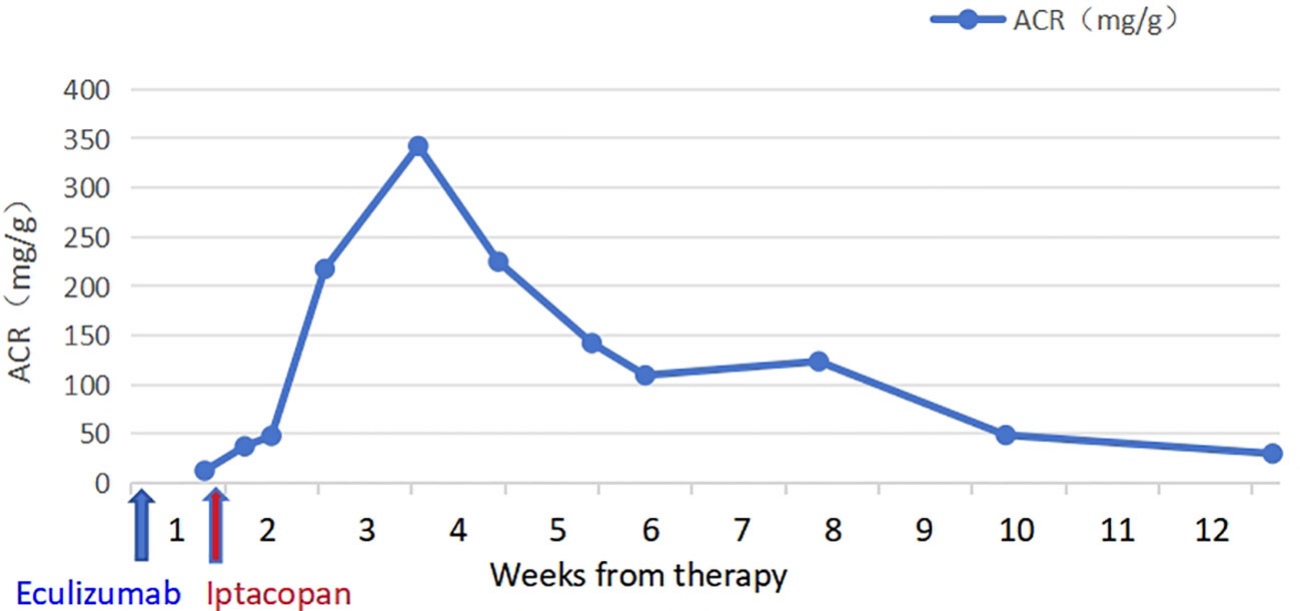
Temporal changes in ACR levels.

**Figure 2:**
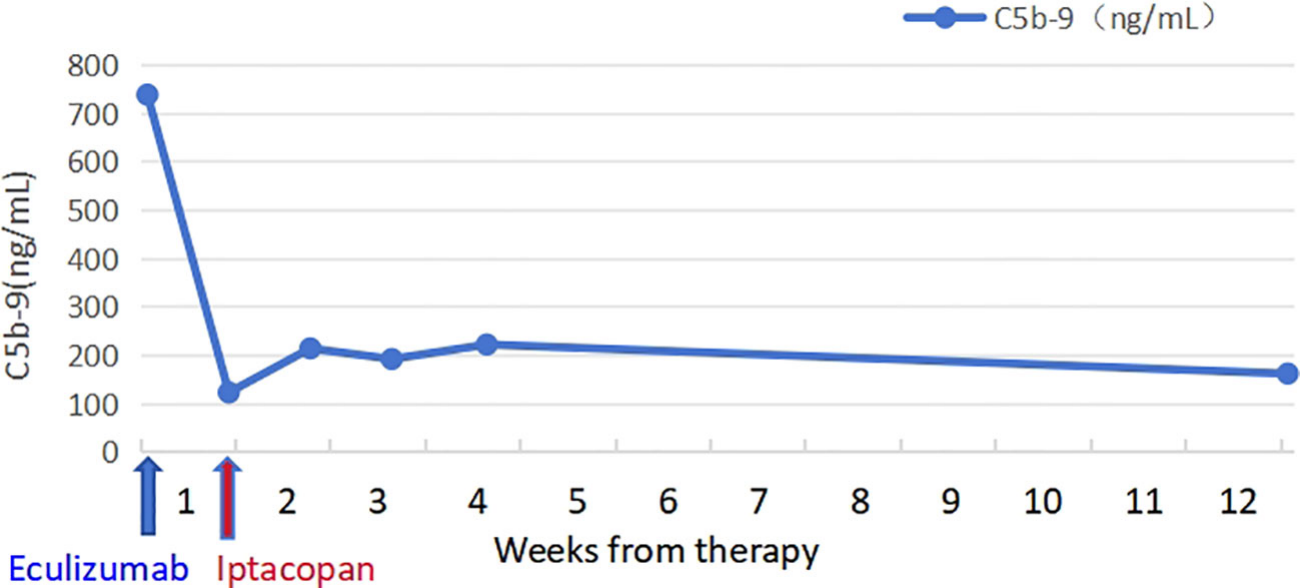
Temporal changes in C5b-9 levels.

**Figure 3:**
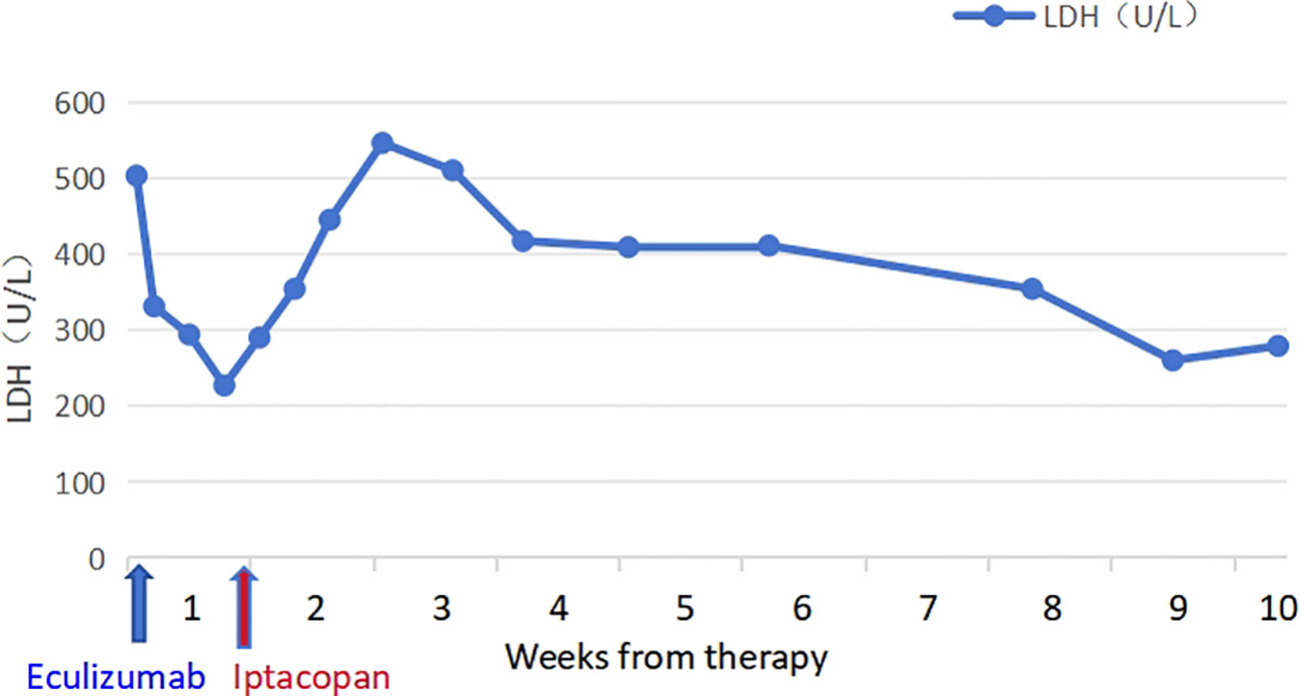
Temporal changes in LDH levels.

**Figure 4:**
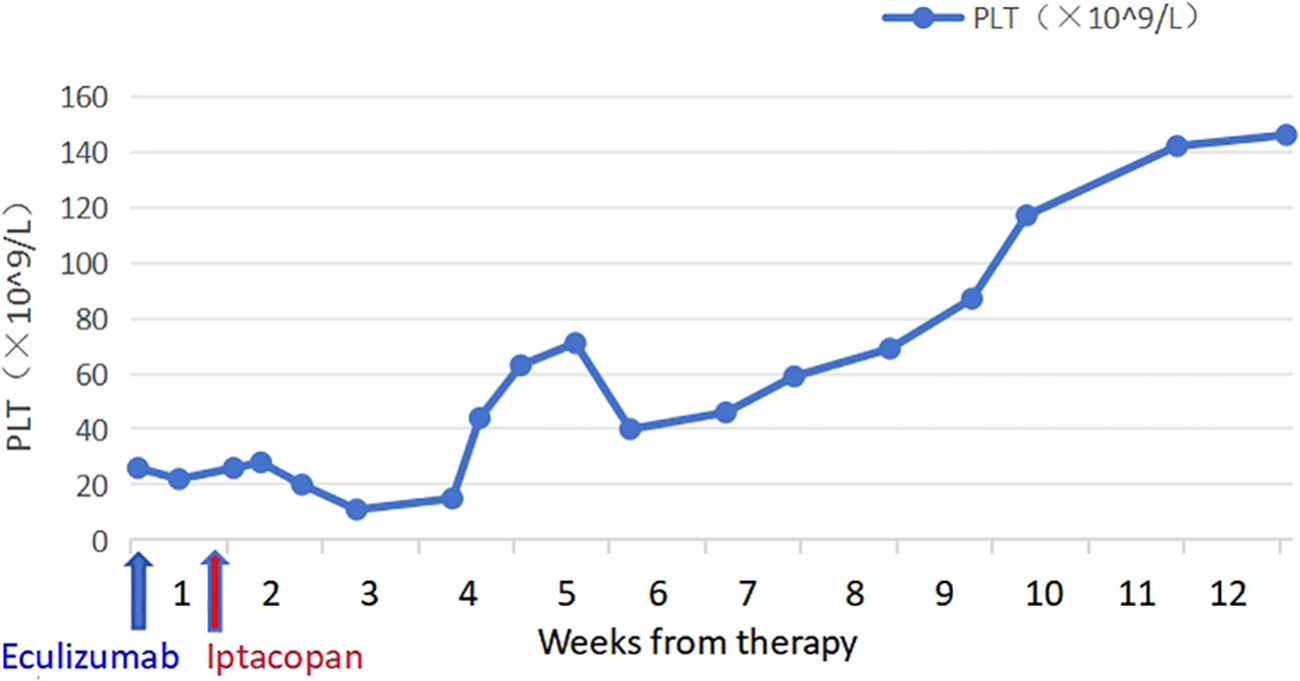
Temporal changes in PLT levels

**Figure 5:**
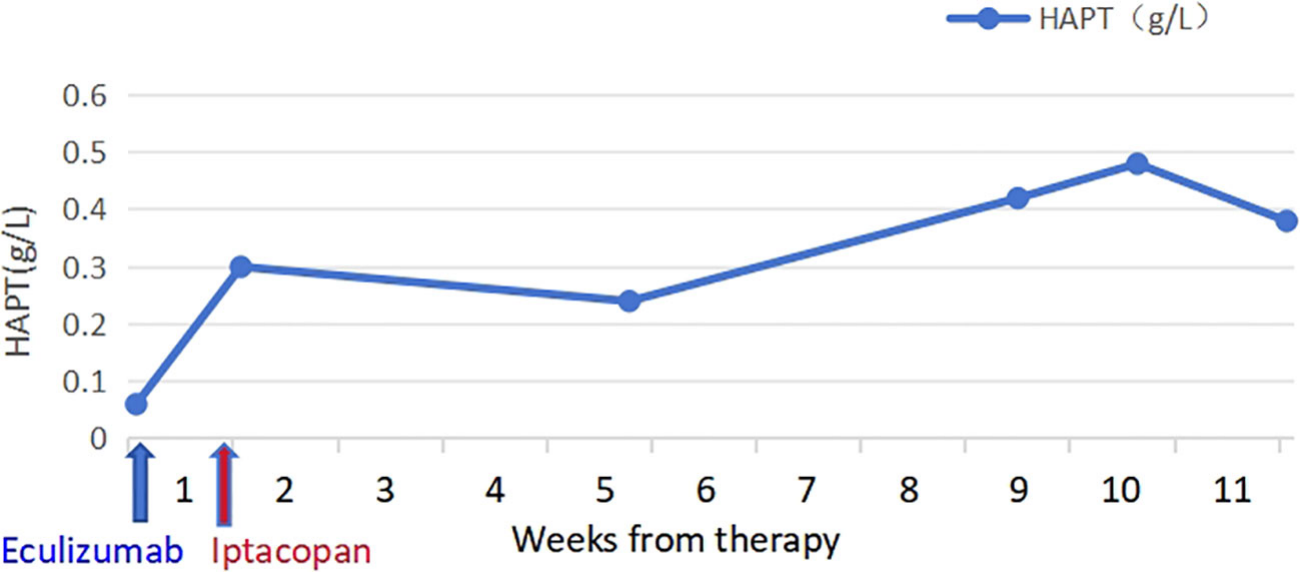
Temporal changes in HAPT levels.

The patient took iptacopan (200 mg, twice daily) for 1 month, during which his condition significantly improved, with clinical indicators gradually returning to normal (**Table 1**). After 10 weeks of iptacopan administration, the patient’s ACR decreased to 29.78 mg/g (**Figure 1**), C5b-9 levels dropped to 163 ng/mL (**Figure 2**), LDH decreased to 279 U/L (**Figure 3**), platelet count increased to 146 × 10^9^/L (**Figure 4**), and haptoglobin recovered to 0.38 g/L (**Figure 5**).

**Table 1:** Clinical laboratory values.

Pre-Iptacopan		Post-Iptacopan				
		2 week	4 week	6 week	8 week	10 week
LDH (U/L),median	417 (331-503)	477 (445-510)	410 (409-411)	382 (354-411)	269 (260-279)	250 (242-258)
ACR (mg/g),median	42.4 (37-47.7)	279.9 (217-342)	183.4 (142-225)	116.4 (109-124)	48.5	29.8
C5b-9 (ng/mL)	431	204	223			163
Haptoglobin (g/L)	0.06	0.3	0.24	0.42	0.48	0.38
PLT (109/L),median	24 (22-26)	18 (15-20)	57 (44-71)	53 (46-59)	78 (69-87)	131 (117-146)

LDH, lactate dehydrogenase; ACR, urinary microalbumin/creatinine ratio; PLT, platelet count.

The patient remained afebrile, and a follow-up chest CT showed resolution of the lung infection. Liver and kidney function normalized, and the patient resumed normal daily urine output and regular bowel movements.

## Discussion

The complement system is closely related to TA-TMA and plays a significant role in its diagnosis, treatment, and prognosis. The C5 monoclonal antibody prevents the formation of the membrane attack complex (MAC) by binding to C5 and regulating the terminal complement pathway, thereby inhibiting complement-mediated intravascular hemolysis (IVH) (8, 12, 13). The C5 antibody inhibitor eculizumab targets C5 and can rapidly control IVH; however, it may leave residual IVH and trigger extravascular hemolysis (EVH). Due to the binding of factor B to C3 (H2O) and its cleavage by factor D, C3 convertase is generated, which cleaves C3 into C3a and C3b. C3b, in turn, forms C3bBbP (C3 convertase) under the action of factor B and factor D, creating an amplification loop of C3b and C3 convertase that rapidly amplifies the complement signal (9). The C5 inhibitor acts on the terminal complement pathway and cannot suppress the amplification loop, resulting in incomplete hemolysis and poor therapeutic outcomes. Additionally, C3b produced after C3 activation deposits on the surface of red blood cells, leading to their destruction in the liver or spleen and causing EVH (10–12, 14, 15). Thus, in cases of residual IVH and EVH caused by persistent proximal complement activation, treatment with eculizumab is often ineffective.

In this case, the patient showed significantly elevated LDH, urine protein/creatinine ratio, and sC5b-9 levels, along with multiple organ failure. This patient was classified as a high-risk TA-TMA patient, with an extremely high mortality rate. Iptacopan is an orally administered complement factor B inhibitor. It binds directly and selectively to factor B, blocking its cleavage by factor D and thereby inhibiting the generation of Ba and Bb fragments. This prevents the formation of C3bBb and halts the activation and amplification cycle of the alternative pathway (16, 17). Iptacopan can effectively inhibit the persistent activation of the proximal complement cascade and compensate for the limitations of C5 antibody inhibitors. Additionally, as an oral formulation, iptacopan is more convenient and better tolerated than the intravenous eculizumab.

After one course of oral iptacopan treatment, the patient’s TA-TMA indicators significantly improved, with a favorable safety profile. While iptacopan has shown promise in the treatment of atypical hemolytic uremic syndrome (aHUS) and paroxysmal nocturnal hemoglobinuria (PNH), its application in TA-TMA remains underexplored. We hope this case provides valuable insight into the treatment of TA-TMA.

## Conclusion

This case report demonstrates the potential efficacy of iptacopan in the treatment of high-risk TA-TMA, particularly in patients with multi-organ dysfunction and severe complement activation. By targeting the proximal complement pathway, iptacopan offers a novel therapeutic approach that may overcome the limitations of existing C5 inhibitors. Further investigation—including larger, well-designed clinical trials—is needed to validate these findings and establish iptacopan as a standard treatment option for TA-TMA.

## Data Availability

The original contributions presented in the study are included in the article/supplementary material. Further inquiries can be directed to the corresponding author.

## References

[1] Jodele S Laskin BL Dandoy CE Myers K EI-Bietar J Davies SM. A new paradigm: Diagnosis and management of HSCT-associated thrombotic microangiopathy as multi-system endothelial injury. Blood Rev. (.(2015) 29:191–204. doi: 10.1016/j.blre.2014.11.001, PMID: 25483393 PMC4659438

[2] Elsallabi O Bhatt VR Dhakal P Foster KW Tendulkar KK. Hematopoietic stem cell transplant-associated thrombotic microangiopathy. Clin Appl Thromb Hemost. (2016) 22:12–20. doi: 10.1177/1076029615598221, PMID: 26239316

[3] Sartain S Shubert S Wu MF Srivaths P Teruya J Krance R. Therapeutic Plasma Exchange does not Improve Renal Function in Hematopoietic Stem Cell Transplantation-Associated Thrombotic Microangiopathy: An Institutional Experience. Biol Blood Marrow Transplant. (2019) 25:157–62. doi: 10.1016/j.bbmt.2018.08.016, PMID: 30144562

[4] Dvorak CC Higham C Shimano KA. Transplant-associated thrombotic microangiopathy in pediatric hematopoietic cell transplant recipients: A practical approach to diagnosis and management. Front Pediatr. (2019) 7:133. doi: 10.3389/fped.2019.00133, PMID: 31024873 PMC6465621

[5] Ma S Bhar S Guffey D Kim RB Jamil N Amos CI. Prospective clinical and biomarker validation of the american society for transplantation and cellular therapy consensus definition for transplantation-associated thrombotic microangiopathy. Transpl Cell Ther. (2023) 29:685.e1–7. doi: 10.1016/j.jtct.2023.08.015, PMID: 37597686 PMC11037887

[6] Okamura H Nakamae H Shindo T Ohtani K Hidaka Y Ohtsuka Y. Early elevation of complement factor ba is a predictive biomarker for transplant-associated thrombotic microangiopathy. Front Immunol. (2021) 12:695037. doi: 10.3389/fimmu.2021.695037, PMID: 34326846 PMC8315095

[7] Sartain S Shubert S Wu MF Wang T Martinez C. The alternative complement pathway activation product Ba as a marker for transplant-associated thrombotic microangiopathy. Pediatr Blood Cancer. (2019) 67:e28070. doi: 10.1002/pbc.28070, PMID: 31774252

[8] Risitano AM Frieri C Urciuoli E Marano L. The complement alternative pathway in paroxysmal nocturnal hemoglobinuria: From a pathogenic mechanism to a therapeutic target. Immunol Rev. (2023) 313:262–78. doi: 10.1111/imr.13137, PMID: 36110036 PMC10087358

[9] Schubart A Anderson K Mainolfi N Sellner H Ehara T Adams CM. Small-molecule factor B inhibitor for the treatment of complement-mediated diseases. Proc Natl Acad Sci USA. (2019) 116:7926–31. doi: 10.1073/pnas.1820892116, PMID: 30926668 PMC6475383

[10] Notaro R Sica M. C3-mediated extravascular hemolysis in PNH on eculizumab: Mechanism and clinical implications. Semin Hematol. (2018) 55:130–5. doi: 10.1053/j.seminhematol.2018.05.014, PMID: 30032749

[11] Notaro R Luzzatto L. Breakthrough hemolysis in PNH with proximal or terminal complement inhibition. N Engl J Med. (2022) 387:160–6. doi: 10.1056/NEJMra2201664, PMID: 35830642

[12] Risitano AM Peffault de Latour R. How we(‘ll) treat paroxysmal nocturnal haemoglobinuria: diving into the future. Br J Haematol. (2022) 196:288–303. doi: 10.1111/bjh.17753, PMID: 34355382 PMC9291300

[13] Harris CL Pouw RB Kavanagh D Sun S Ricklin D. Developments in anti-complement therapy; from disease to clinical trial. Mol Immunol. (2018) 102:89–119. doi: 10.1016/j.molimm.2018.06.008, PMID: 30121124

[14] Risitano AM Notaro R Marando L Serio B Ranaldi D Seneca E. Complement fraction 3 binding on erythrocytes as additional mechanism of disease in paroxysmal nocturnal hemoglobinuria patients treated by eculizumab. Blood. (2009) 113:4094–100. doi: 10.1182/blood-2008-11-189944, PMID: 19179465

[15] Lin Z Schmidt CQ Koutsogiannaki S Ricci P Risitano AM Lambris JD. Complement C3dg-mediated erythrophagocytosis: implications for paroxysmal nocturnal hemoglobinuria. Blood. (2015) 126:891–4. doi: 10.1182/blood-2015-02-625871, PMID: 26082452 PMC4536542

[16] Jang JH Wong L Ko BS Yoon SS Li K Baltcheva I. Iptacopan monotherapy in patients with paroxysmal nocturnal hemoglobinuria: a 2-cohort open-label proof-of-concept study. Blood Adv. (2022) 6:4450–60. doi: 10.1182/bloodadvances.2022006960, PMID: 35561315 PMC9636331

[17] Peffault de Latour R Röth A Kulasekararaj AG Han B Scheinberg P Maciejewski JP. Oral iptacopan monotherapy in paroxysmal nocturnal hemoglobinuria. N Engl J Med. (2024) 390:994–1008. doi: 10.1056/NEJMoa2308695, PMID: 38477987

